# Effects of Fluoxetine on Hippocampal Neurogenesis and Neuroprotection in the Model of Global Cerebral Ischemia in Rats

**DOI:** 10.3390/ijms19010162

**Published:** 2018-01-05

**Authors:** Marina Khodanovich, Alena Kisel, Marina Kudabaeva, Galina Chernysheva, Vera Smolyakova, Elena Krutenkova, Irina Wasserlauf, Mark Plotnikov, Vasily Yarnykh

**Affiliations:** 1 Laboratory of Neurobiology, Research Institute of Biology and Biophysics, Tomsk State University, 36, Lenina Ave., Tomsk 634050, Russia; kisell.alena@gmail.com (A.K.); kmsra08@gmail.com (M.K.); len--k@yandex.ru (E.K.); i-2811-na@yandex.ru (I.W.); yarnykh@uw.edu (V.Y.); 2 E.D. Goldberg Institute of Pharmacology and Regenerative Medicine, 3, Lenina Ave., Tomsk 634028, Russia; bona2711@mail.ru (G.C.); light061265@mail.ru (V.S.); mbp2001@mail.ru (M.P.); 3 Department of Radiology, University of Washington, Seattle, WA 98195, USA

**Keywords:** global brain ischemia, neurogenesis, fluoxetine, hippocampus, dentate gyrus, inflammation, CA1, antidepressants, neural stem cells, rats

## Abstract

A selective serotonin reuptake inhibitor, fluoxetine, has recently attracted a significant interest as a neuroprotective therapeutic agent. There is substantial evidence of improved neurogenesis under fluoxetine treatment of brain ischemia in animal stroke models. We studied long-term effects of fluoxetine treatment on hippocampal neurogenesis, neuronal loss, inflammation, and functional recovery in a new model of global cerebral ischemia (GCI). Brain ischemia was induced in adult Wistar male rats by transient occlusion of three main vessels originating from the aortic arch and providing brain blood supply. Fluoxetine was injected intraperitoneally in a dose of 20 mg/kg for 10 days after surgery. To evaluate hippocampal neurogenesis at time points 10 and 30 days, 5-Bromo-2′-deoxyuridine was injected at days 8–10 after GCI. According to our results, 10-day fluoxetine injections decreased neuronal loss and inflammation, improved survival and functional recovery of animals, enhanced neurogenesis, and prevented an early pathological increase in neural stem cell recruitment in the subgranular zone (SGZ) of the hippocampus without reducing the number of mature neurons at day 30 after GCI. In summary, this study suggests that fluoxetine may provide a promising therapy in cerebral ischemia due to its neuroprotective, anti-inflammatory, and neurorestorative effect.

## 1. Introduction

Fluoxetine, a known selective serotonin reuptake inhibitor, is widely used in clinical practice for treatment of depressive and anxiety-like disorders. In addition, recent studies have shown the capability of fluoxetine to enhance neurogenesis and neuronal survival [1,2,3,4,5,6,7,8,9,10,11]. It has been demonstrated that fluoxetine increases neurogenesis in both normal [1,2,3,4,5,6] conditions and after ischemic injury [7,8,9,10].

In the normal adult brain, neurogenesis continuously occurs in specific anatomical regions, including the subventricular zone (SVZ) near the lateral ventricles and subgranular zone (SGZ) of the dentate gyrus (DG) of the hippocampus [12]. Numerous studies have shown significant changes in neurogenesis in pathological conditions, particularly, in the models of cerebral ischemia [10,12,13,14,15,16,17,18,19,20,21,22,23,24]. Cerebral ischemia typically results in an increase in neurogenesis in the SVZ and DG [12,13,14,15,16,17,18,19,20,21,22,23]. The well-standardized and widely used model of middle cerebral artery occlusion (MCAO) causes an enhancement of neurogenesis, mostly, in the SVZ and the migration of a large number of neuronal progenitors to the damaged area, where they pass the stage of maturation and integrate into neural networks [12,13,19,20,21,22]. Several studies have also showed increased cell proliferation in the DG [20,21] and even in non-neurogenic cortex regions [23].

Fluoxetine treatment was demonstrated to improve functional recovery after focal ischemia including neurological scores [25], spatial memory [7,26], and sensorimotor functions [25]. It was shown histologically that fluoxetine decreased the infarct size [26] in the MCAO model and demonstrated a neuroprotective effect [25,27,28].

While the above results were derived with the use of focal brain ischemia models, little is known about the effect of fluoxetine in global cerebral ischemia (GCI). Such a condition occurs if the brain completely loses blood supply, and it is clinically related to the transient cardiac arrest. Several GCI models have been developed including the two-vascular model of forebrain ischemia in gerbils [14,16,24], the two-vascular cerebral ischemia model in rats with hypotension [15], murine chemically-induced cardiac arrest model [29], and a recent three-vessel rat occlusion model [30,31]. As compared to focal ischemic infarct models, GCI models are less standardized and used less frequently. A contribution to post-ischemic neurogenesis for each of two different neurogenic niches (SVZ and DG) strongly depends on the model of cerebral ischemia. In general, studies on GCI models [14,15,16] suggest of an increase in neurogenesis in the DG in the early period with a peak of proliferation at day 10 after surgery. A few investigations of the effect of fluoxetine in GCI have been published to date [10,27,28,29]. The neuroprotective effect of high-dose fluoxetine has been found in the caudate-putamen but not in the hippocampus in the early period (3 days) after the ischemic event in the murine cardiac arrest model [29]. The studies performed on the two-vessel GCI model in gerbils [28] and mice [27] have shown neuroprotective effect of fluoxetine in the hippocampus. Preliminary results of our study [10] based on the rat three-vessel occlusion GCI model [30,31] have shown a decrease in the number of immature neurons in the DG after fluoxetine treatment at later observation times (30 days). Clearly, these limited observations do not provide a complete picture of a potential therapeutic effect of fluoxetine in GCI and warrant further studies. The present study aimed to comprehensively evaluate neuroprotective and neurogenic potential of fluoxetine treatment in GCI including quantitative assessment of neuronal loss and recovery, NSC proliferation, and production and maturation of neurons in the neurogenic zones at different time points after ischemic injury.

## 2. Results

### 2.1. Survival and Neurological Deficit

Survival rates and neurological scores in the animal groups after surgery are summarized in Table 1. At 10 days after GCI only 11 (40.7%) of 27 rats from positive controls survived; in 30 days after surgery the survival rate decreased to 37%. The highest mortality rate was observed during the first hour after total cerebral ischemia modeling and survived animals showed a severe CNS deficit, such as areflexia, spastic paralysis, and muscle tension. The severe neurological deficit continued up to day 10 after GCI. At day 30 neurological scores of positive controls slightly decreased but not significantly compared with an earlier time point. Fluoxetine treatment improved survival rates both at days 10 and 30 after GCI, up to 61.1%.

Neurological deficit in the fluoxetine-treated animals was also less severe than in positive controls. In 10 days after GCI, neurological scores did not differ from positive controls significantly. However, by day 30 after GCI, neurological deficit in the fluoxetine-treated animals decreased significantly compared with positive controls and did not differ from the sham-operated animals. In the sham-operated group, the surgery did not cause death of rats or impairment of neurological function.

### 2.2. Fluoxetine Reduces Neuronal Loss in the Hippocampus

**Figure 1:**
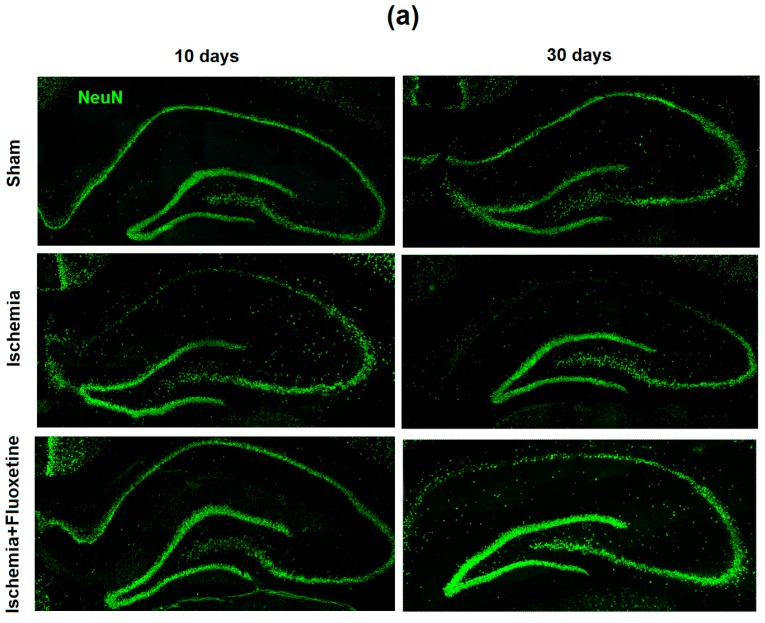

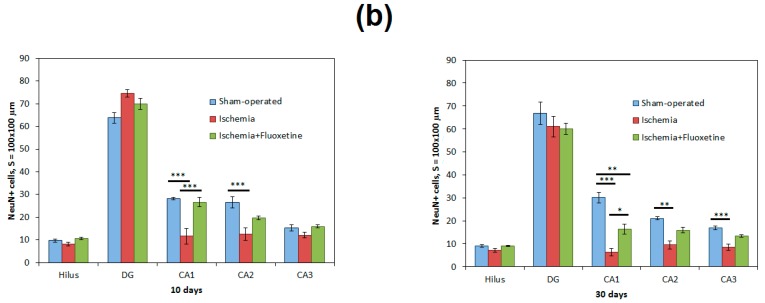
Fluoxetine effect on neuronal loss in the hippocampus after GCI. (**a**) Micrographs of the whole hippocampus of the sham-operated animals, positive controls, and fluoxetine-treated animals after GCI at days 11 and 31 after surgery, 10× magnification. Brain sections were stained with NeuN. (**b**) Comparison of total number of neurons in hippocampal regions per 100 × 100 µm^2^ between the groups. Significant differences between the groups, according to ANOVA after Bonferroni’s correction for multiple comparisons: * *p* < 0.05, ** *p* < 0.01, *** *p* < 0.001.

Analysis of the microphotographs of brain sections obtained at days 11 and 31 after surgery showed a substantial loss of hippocampal CA1 neurons after ischemia (Figure 1a). Quantitative analysis confirmed a significant, more than two-fold, decrease in the neuronal density in CA1, CA2, and CA3 (for time point 30 days), but not in the DG and hilus (Figure 1b and Table S1). The 10-day treatment with fluoxetine prevented significant neuronal loss at the time point of 11 days after GCI, but did not prevent partial reduction in the number of neurons at the time point of 31 days after GCI (Figure 1b and Table S1). The total number of neurons in CA1 in the fluoxetine-treated animals did not differ from sham-operated at day 11 after GCI. However, at day 31 after surgery, the number of neurons in CA1 was significantly lower than that in sham-operated but significantly higher compared to positive controls.

### 2.3. Fluoxetine Effect on Ischemia-Induced Proliferation in the Hippocampus

**Figure 2:**
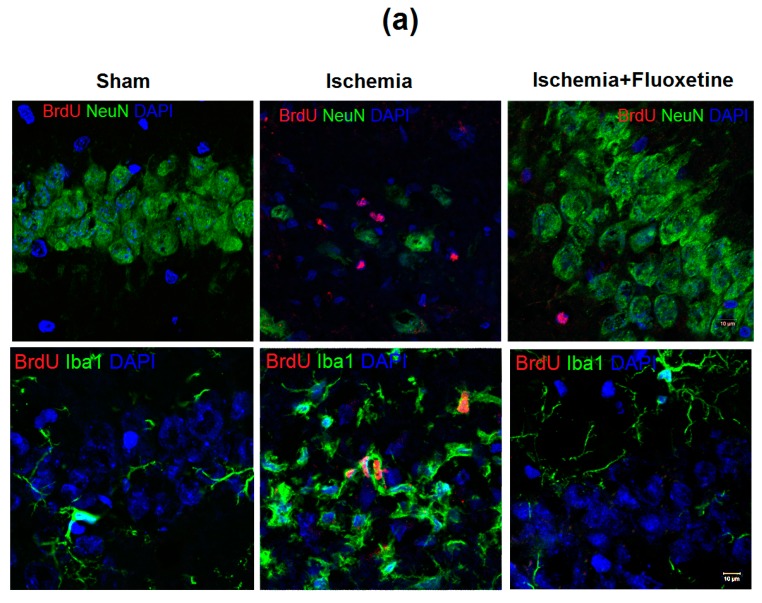

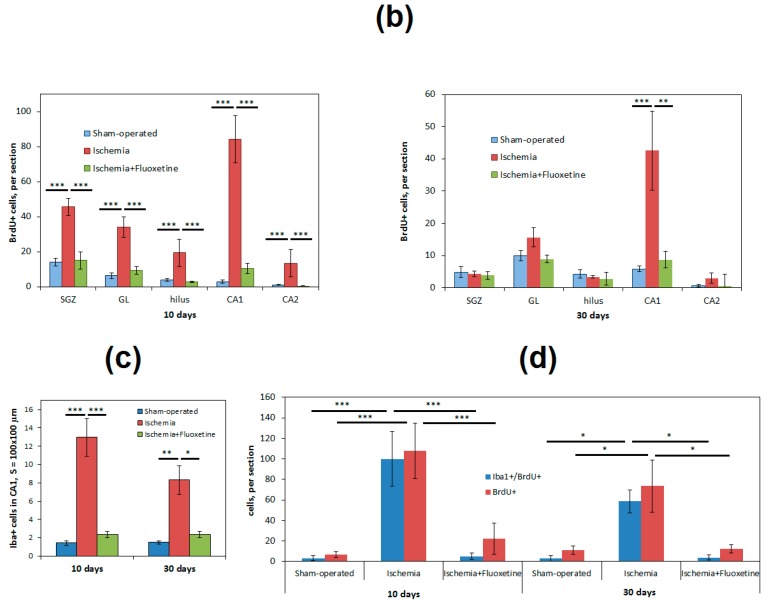
Fluoxetine effect on cell proliferation and inflammation. (**a**) Micrographs of the CA1 field of the hippocampus of the sham-operated animals, positive controls, and fluoxetine-treated animals at day 11 after GCI. Brain sections were stained with BrdU, NeuN and DAPI (top row) and BrdU, Iba1 and DAPI, 63× magnification; (**b**) Comparison of BrdU+ cells in hippocampal regions between the groups at days 11 (**b**, left) and 31 (**b**, right) after surgery; (**c**) Comparison of Iba1+ cells in the CA1 field between the groups at days 11 and 31 after surgery; (**d**) The number of Iba1\BrdU double-positive cells in the CA1 field at days 11 and 31 after surgery compared to the total number of BrdU+ cells. Significant differences between the groups, according to ANOVA after Bonferroni’s correction for multiple comparisons: * *p* < 0.05, ** *p* < 0.01, *** *p* < 0.001.

GCI caused a profound increase in cell proliferation in all hippocampal regions compared with the sham-operated animals at day 11 after surgery (Figure 2 and Table S1). The most prominent, more than 20-fold, growth of cell proliferation was observed in the CA1 hippocampal field. Most of BrdU+ cells were not colocalized with NeuN labeling (Figure 2a and Table S1). An increase in cell proliferation in the subgranular zone (SGZ) of the DG was considerable too.

As for positive controls, the number of BrdU+ cells in the SGZ was three times higher than that in the sham-operated rats. ANOVA with post-hoc tests confirmed a substantial increase in cell proliferation at day 11 after GCI in all hippocampal regions except for the CA2 field. At day 31 after GCI, cell proliferation in positive controls remained also significantly increased in the CA1 field.

The fluoxetine treatment completely prevented the ischemia-induced increase in cell proliferation in the hippocampus. At both days 11 and 31, the level of cell proliferation in the fluoxetine-treated group was significantly lower than that in the non-treated animals after GCI and did not differ from the control level.

### 2.4. Anti-Inflamatory Effect of Fluoxetine

GCI caused extensive inflammation in the hippocampus, which was mostly noticeable as microglial proliferation in the CA1 field (Figure 2a and Table S1). The morphology of microglial cells changed in a characteristic way including enlargement of soma and reduction of cell processes. At day 11 after surgery, the number of microglial cells increased more than 10-fold in positive controls, whereas the fluoxetine-treated animals did not show significant distinctions compared with the sham-operated group (Figure 2c and Table S1). At day 31 after surgery, the number of microglial cells still remained significantly higher in the sham-operated and fluoxetine-treated groups. Analysis of double labeling Iba1\BrdU cells (Figure 2d) showed that most BrdU+ cells were colocalized with Iba1 expression. The number of double-positive BrdU\Iba1 cells in the CA1 field was significantly higher in positive controls at days 11 and 31 after surgery compared with the sham-operated and fluoxetine-treated groups.

### 2.5. Fluoxetine Effect on NSC Recruitment in the DG of the Hippocampus

**Figure 3:**
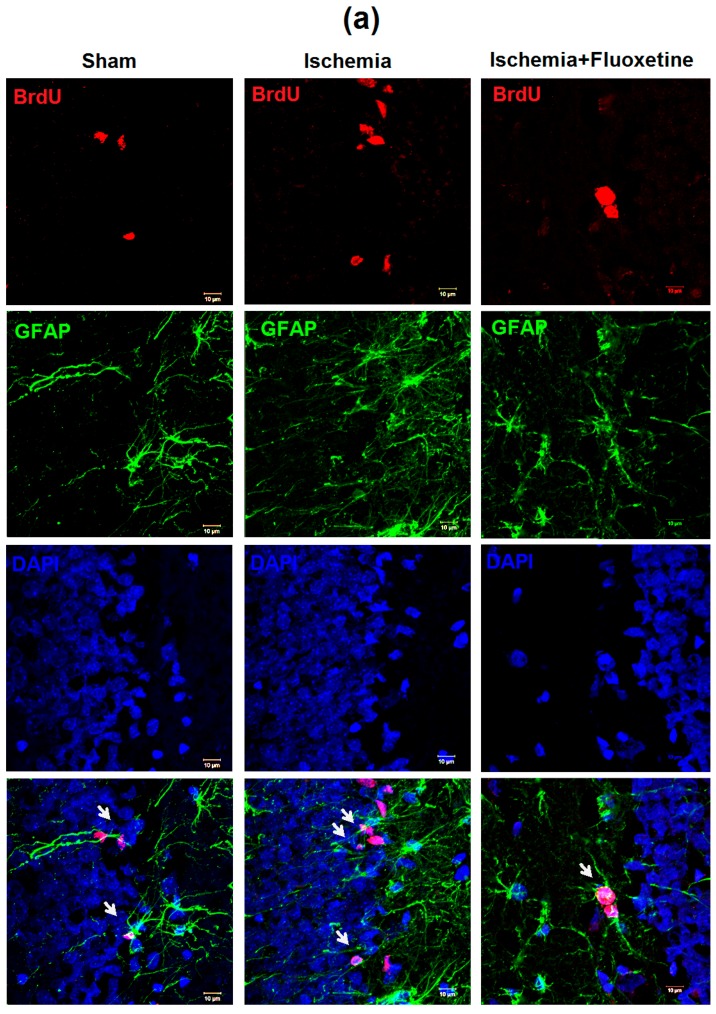

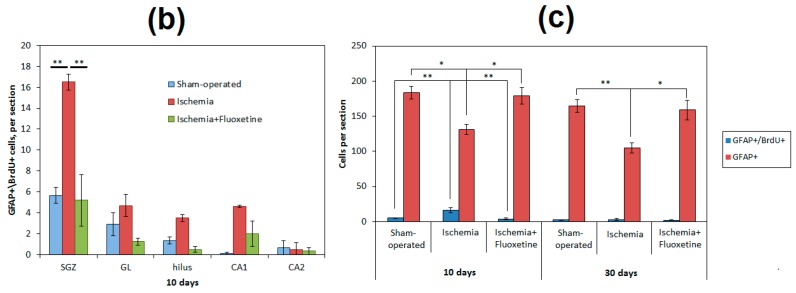
Fluoxetine effect on neural stem cell recruitment in the DG. (**a**) Micrographs of the DG of the hippocampus in the sham-operated animals, positive controls, and fluoxetine-treated animals at day 11 after GCI. Brain sections were stained with BrdU, GFAP and DAPI, 63× magnification. NSCs are indicated by arrows; (**b**) Comparison of GFAP+\BrdU+ cells in hippocampal regions between the groups at day 11 (**b**) after surgery; (**c**) Comparison of GFAP+\BrdU− (quiescent NSC) cells and GFAP+\BrdU+ (recruited NSC) cells in the SGZ between the groups at days 11 and 31 after surgery. Significant differences between the groups, according to ANOVA after Bonferroni’s correction for multiple comparisons: ** *p* < 0.01, * *p* < 0.05.

Neural stem cell (NSC) recruitment was evaluated as a number of GFAP+\BrdU+ cells in the SGZ of the DG at day 11 after surgery. Whereas BrdU was injected from days 8 to 10 after GCI, only this time point allowed assessing the recently proliferated NSCs. The ischemia increased the number of recruited NSCs more than twice (Figure 3 and Table S1). At the same time, the number of NSCs (GFAP+ cells of radial glia-like morphology) in positive controls was significantly lower than that in sham-operated animals at both time points. On average, 12.6% of NSCs in positive controls were colocalized with BrdU, whereas only 2.8% and 2.1% of NSCs were recruited in the sham-operated and fluoxetine-treated animals correspondingly. Fluoxetine injections significantly reduced post-ischemic elevation of NSC recruitment to the control level. The number of NSCs in the fluoxetine-treated animals also did not differ from the sham-operated group.

### 2.6. Fluoxetine Effect on Neurogenesis in the DG of the Hippocampus

**Figure 4:**
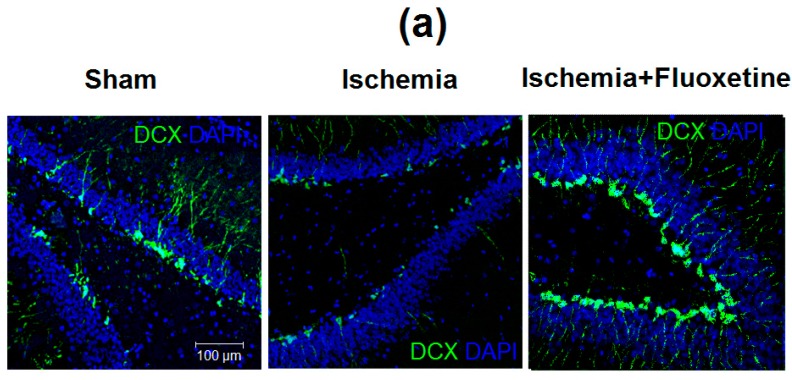

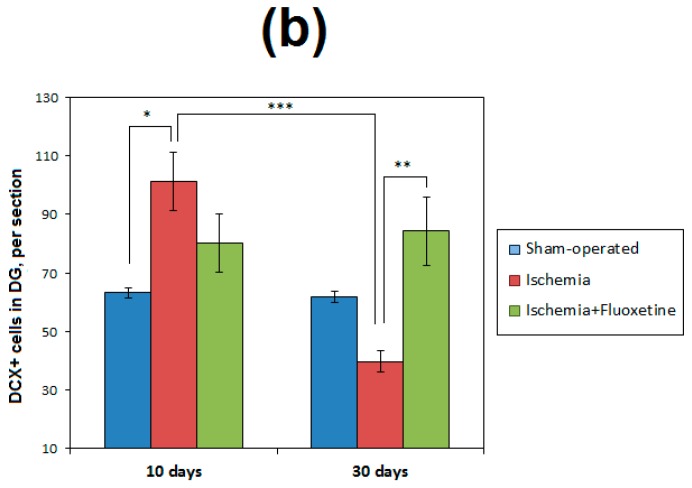
Fluoxetine effect on neurogenesis in the DG. (**a**) Micrographs of the DG of the hippocampus in the sham-operated animals, positive controls, and fluoxetine-treated animals at day 31 after GCI. Brain sections were stained with DCX and DAPI, 20× magnification; (**b**) Comparison of the number of DCX+ cells in the SGZ between the groups and time points. Significant differences between the groups according to ANOVA after Bonferroni’s correction for multiple comparisons: *** *p* < 0.001, ** *p* < 0.01, * *p* < 0.05.

The number of neural progenitors (DCX+ cells) in the DG was significantly increased in the positive controls compared with the sham-operated group on the 11th day after GCI and then demonstrated an about 2.5-fold drop on the 31st day, being significantly lower than the 11th day value (Figure 4 and Table S1). Neurogenesis in the sham-operated and fluoxetine-treated animals was similar at both time points with a trend of an elevated DCX+ cell count in the fluoxetine group. The fluoxetine-treated animals also showed an increased level of neurogenesis at day 31 after surgery as compared to the non-treated GCI group.

**Figure 5:**
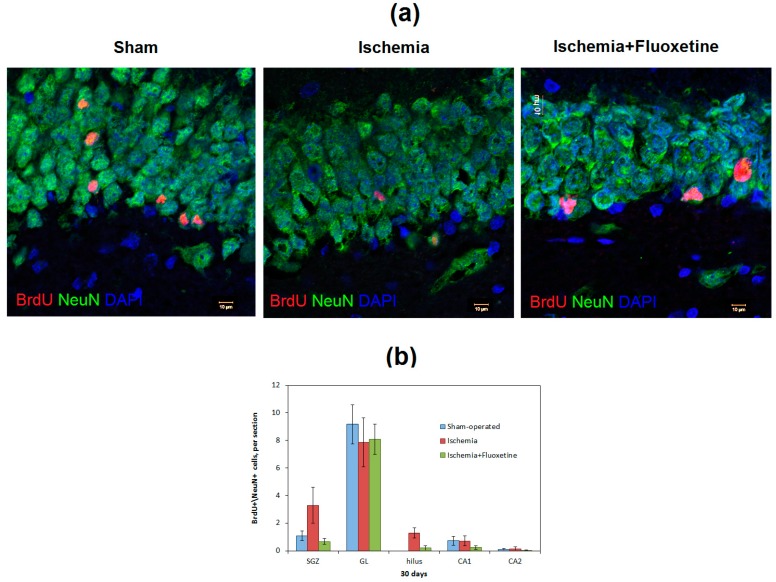
Fluoxetine effect on maturation of newborn neurons. (**a**) Micrographs of the DG of the hippocampus in the sham-operated animals, positive controls, and fluoxetine-treated animals at day 31 after GCI. Brain sections were stained with BrdU, NeuN and DAPI, 63× magnification; (**b**) Comparison of the number of NeuN+\BrdU+ cells in the regions of the hippocampus between the groups. No significant differences between the groups.

Mature neurons were detected by colocalization of BrdU and NeuN labeling at day 31 after GCI. Both positive controls and the fluoxetine-treated animals did not show significant changes in the number of mature neurons (NeuN+\BrdU+ cells) compared with the sham-operated animals (Figure 5 and Table S1).

## 3. Discussion

**Figure 6:**
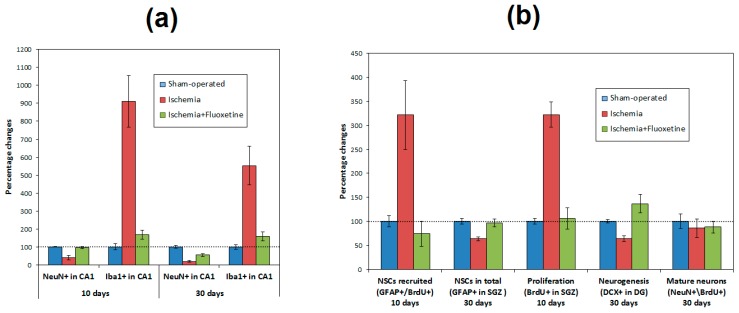
Summary of fluoxetine effects on neuronal loss, inflammation, and neurogenesis in the DG after GCI. (**a**) Mean percentage changes in the number of NeuN+ and Iba1+ cells in the CA1 field at days 11 and 31 after GCI; (**b**) Mean percentage changes in the number of recruited NSCs, and proliferation at day 11 after surgery, and in total number of NSCs, neurogenesis, and mature neurons at day 31 after surgery.

The key result of our study is the demonstration of the neuroprotective and anti-inflammatory effect of fluoxetine in the rat GCI model (Figure 6a). This effect manifested as a significant reduction in the neuronal death in the hippocampus under fluoxetine treatment in the 10-day window after the ischemic event. This effect was accompanied by the improvement of functional recovery and neurogenesis in the hippocampus. Our results also indicate that fluoxetine treatment during the first 10 days after GCI improved survival rate and neurological scores of the experimental animals. However, the effect of fluoxetine weakened after treatment discontinuation, thus indicating that a longer treatment may be needed to achieve therapeutically relevant outcomes.

Our results are in overall agreement with numerous studies that found the neuroprotective and anti-inflammatory action of fluoxetine and its positive effects on functional outcome after ischemia in rodents [7,8,9,25,26,27,28,29]. However, several studies did not reveal neuroprotection caused by fluoxetine treatment [32,33]. A variety of doses (5–50 mg/kg), as well as timing of fluoxetine administration (from pre-surgery to post-ischemia period with different duration) could explain these discrepancies. The majority of the above studies were performed on focal ischemia models, which are characterized by the primary neuronal loss in the cortex and striatum [12,13,19,20,21,22,23], whereas the model of global ischemia causes neuronal damage to the hippocampus [10,14,15,16,24].

A few studies on the effect of fluoxetine in GCI have been done to date. The neuroprotective effect of fluoxetine in a dose of 10 mg/kg in 72 h after cardiac arrest in mice was found in the striatum but not in the hippocampus [29]. Kim et al. [28] observed neuroprotective activity of high-dose fluoxetine in the hippocampus in the two-vessel GCI model in gerbils. A similar neuroprotective effect of fluoxetine was described in [27]. Our study also demonstrated the neuroprotective effect on the hippocampus.

An important observation of our study is the dramatic reduction in ischemia-induced cell proliferation after fluoxetine administration, most noticeable in the damaged non-neurogenic CA1 field of the hippocampus. Fluoxetine restored global cell proliferation to the level of sham-operated controls at both 10- and 30-day time points. Our results showed that the observed effect is related to anti-inflammatory activity as indicated by a reduction in microglial activation. Activated microglia represent the most abundant proliferating cell type in the ischemic lesion in accordance with the results of other studies [34]. The observed anti-inflammatory effect of fluoxetine is also in agreement with earlier studies [25,27,35].

Another important finding of this study is an impact of fluoxetine treatment on hippocampal neurogenesis. Although we did not find a significant increase in the number of mature neurons, the efficiency of new neuron development in terms of cellular supply and survival was significantly higher in the fluoxetine-treated animals than in the non-treated positive controls. Recent studies have convincingly demonstrated a restriction of the NSC pool in the SGZ of the hippocampus and its depletion during aging [36]. Our previous study [10] showed that an impairment of neurogenesis in 30 days after GCI was restored by fluoxetine treatment. The present study demonstrates an almost 3-fold increase in stem cell recruitment in the SGZ at day 11 after GCI and further depletion of the total NSC pool in the hippocampus. The initial post-ischemic elevation of neurogenesis was followed by a decline in the production of new neurons. Fluoxetine treatment also raised the level of neurogenesis without further decrease in neuronal production and additional NSC recruitment. Our findings are supported by the studies that revealed specific amplifying impact of fluoxetine on symmetric divisions of early neural progenitors in the DG [3]. Our study also showed that in the fluoxetine-treated animals the NSC pool was not depleted and the level of neurogenesis remained increased in one month after GCI.

Intensification of neurogenesis or improved survival of new neurons after fluoxetine treatment has been previously shown in both normal [1,2,3,4,5,6] and post-ischemic conditions in the focal ischemia model [7,8,9]. In post-ischemic conditions, the effect of fluoxetine on neurogenesis was associated with an improved survival of new neurons rather than the increased number of newborn neurons [7]. Our results, however, show a cooperative effect of intensification of neurogenesis, neuroprotection, and anti-inflammatory action in the fluoxetine-treated animals. Although we revealed no increase in the number of mature neurons after fluoxetine treatment, it should be noted that an elevation of the number of immature neurons at day 31 after GCI could manifest as an elevation of the number of mature neurons at later observation times. Our observations did not provide any evidence of significant reparative neurogenesis in non-neurogenic hippocampal regions including CA1 and CA2.

## 4. Materials and Methods

### 4.1. Animals and Housing

The study was performed on adult male Wistar rats weighing 250–300 g (*n* = 70; 32 rats survived after surgery) obtained from the vivarium of the E.D. Goldberg Institute of Pharmacology and Regenerative Medicine. Experiments were carried out in accordance with the rules adopted by the European Convention for the Protection of Vertebrate Animals used for Experimental and other Scientific Purposes. The study was approved by the Animal Care and Use Committee at the E.D. Goldberg Institute of Pharmacology and Regenerative Medicine (protocol #22032012).

Animals were housed in groups of seven animals per cage with about 300 cm^2^ per animal under standard conditions (12/12 h light/dark cycle, temperature of 22 ± 2 °C, humidity of 60%). Standard rodent chow (PK-120-1, Laboratorsnab Ltd., Moscow, Russia) and water were provided ad libitum.

### 4.2. Experimental Design

Animals were randomly divided into three groups: sham-operated animals (*n* = 10); positive controls, which underwent GCI by transient occlusion (*n* = 10); and animals after ischemia with fluoxetine treatment (*n* = 11). The experimental design is schematically shown in Figure 6a.

Acute global cerebral ischemia was induced according to the new 3-vessel model, as detailed elsewhere [30,31]. Briefly, rats were anesthetized with chloral hydrate in a dose of 450 mg/kg intraperitoneally (Sigma-Aldrich Chemical Co., St. Louis, MO, USA) and placed on a homeothermic blanket (Temperature Control Unit HB 101/2, Letica Scientific Instruments, Barcelona, Spain) in a supine position. The body temperature was maintained at 37 °C. Cerebral blood flow was completely interrupted for 7 min by ligation of the major arteries supplying the brain (truncus brachiocephalicus, arteria subclavia sinistra, and arteria carotis communis sinistra). Access to arteria carotis communis sinistra was implemented through the ventral surface of the neck, while truncus brachiocephalicus and arteria subclavia sinistra were reached through the first intercostal space, bypassing the pleural cavity to avoid pneumothorax. The animals were intubated through the oral cavity. The same surgery was performed in sham-operated rats but without ligation of blood vessels.

Animals from the fluoxetine-treated group were injected with fluoxetine (Sigma-Aldrich Chemical Co.) intraperitoneally in a dose of 20 mg/kg, that showed a positive effect in previous studies [7,9], daily during the first hour after surgery and 9 days after. Animals in the positive control and sham-operated groups were injected with 0.9% saline. All animals received injections of 5-Bromo-2′-deoxyuridine (BrdU, Sigma-Aldrich Chemical Co.) in a dose of 50 mg/kg [24] for three consecutive days, from day 8 to day 10 after surgery. At days 11 and 31 after surgery, neurological deficit was evaluated with the Stroke-index McGraw scale [30,37]. Half of animals from each group were euthanized at days 11 and 31 after surgery by transcardial perfusion with 4% paraformaldehyde under ether anesthesia. The brains were removed, fixed overnight with 4% paraformaldehyde solution, cryoprotected in sucrose phosphate buffer (24 h in 10% and 24 h in 20% solutions, respectively) at 4 °C, frozen in liquid nitrogen, and stored at −80 °C for further immunohistochemical studies.

### 4.3. Immunochemistry

Coronal brain sections with 10 µm thickness were prepared using an HM525 cryostat (Thermo Fisher Scientific, Walldorf, Germany). Brain locations for immunohistochemical analysis were defined from −2.64 mm to −3.60 mm from bregma according to a rat brain atlas [38]. For detection of immature neurons (DCX+ cells), sections were stained with the primary (Doublecortin (C-18): sc-8066, Santa Cruz Biotechnology, Inc., Santa Cruz, CA, USA) and secondary (Donkey anti goat AlexaFlour 488 (705-545-147), Jackson ImmunoResearch Laboratories, West Grove, PA, USA) antibodies and then covered with mounting medium with DAPI (4′,6-diamidino-2-phenylindole). For double immunofluorescence detection of BrdU and NeuN, at first the sections were incubated in 2N HCl at 37 °C for 15 min to denature DNA. After washing in phosphate buffer solution (PBS) the sections were incubated with the primary antibodies for BrdU (mouse-anti-BrdU (IIB5), sc-32323, Santa Cruz Biotechnology) and NeuN (rabbit-anti-NeuN, ABN78, Merck Millipore, Bedford, MA, USA) in PBS overnight at 4 °C, then incubated with the secondary antibodies (Donkey-anti-rabbit AlexaFlour 488, 711-545-152 and Donkey-anti-mouse AlexaFlour 594, 715-585-150, Jackson ImmunoResearch Laboratories, West Grove, PA, USA) and finally covered with mounting medium with DAPI. For double immunolabeling for BrdU/GFAP and BrdU/Iba1 the same above protocol was implemented except for using the primary antibodies for GFAP (rabbit-anti-GFAP, ab7260, Abcam, Cambridge, MA, USA) and Iba1 (rabbit-anti-Iba1, 019-19741, Wako Pure Chemical, Richmond, VA, USA).

For each animal, at least 4 microphotographs of the whole hippocampus of both the left and right hemispheres were obtained. Photographing was performed using an Axio Imager Z2 (Carl Zeiss, Oberkochen, Germany) microscope and AxioVision 4.8 (Carl Zeiss) software with a MozaiX module. Additionally, colocalization for triple (NeuN, BrdU, DAPI and GFAP, BrdU, DAPI) labeling was specified using a laser confocal microscope LSM 780 NLO (Carl Zeiss).

### 4.4. Image Processing

**Figure 7:**
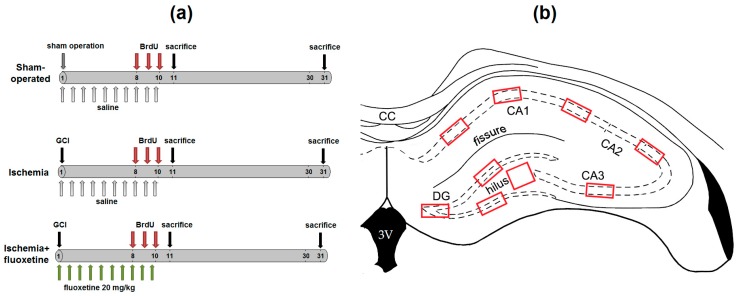
(**a**) Experimental design of the study; (**b**) Scheme of NeuN+ cells counting to quantify severity of the ischemic lesion in experimental animals.

Calculation of NeuN+, BrdU+, NeuN+\BrdU+, GFAP+\BrdU+, Iba1+\BrdU+ cells was carried out using ImageJ software (National Institutes of Health, Bethesda, MD, USA).To quantify the severity of the ischemic lesion, the neuronal loss was evaluated by visual calculation of NeuN+ cells in the DG, hilus, CA1, CA2, and CA3 fields of the hippocampus according to the scheme presented in Figure 7b. To quantify microglial activation, the number of Iba1+ cells was calculated in the CA1 field according to the same scheme. The cells were counted within regions of interest of standard size (200 × 200 µm^2^ for the hilus, 100 × 200 µm^2^ for other regions) and then were normalized to 100 × 100 µm^2^ area. The number of NeuN+\BrdU+, GFAP+\BrdU+, and BrdU+ cells was counted separately in the SGZ and GL of the DG, as well as in the hilus, CA1, and CA2 fields of the hippocampus. The number of Iba1+\BrdU+ and BrdU+ cells was counted in the CA1 field of hippocampus. The number of DCX+ cells was counted in the DG of the hippocampus. GFAP+\BrdU+ cells in the SGZ of the DG were considered as NSCs recruited from day 8 to day 10 after surgery when BrdU was injected. The number of GFAP+ cells of specific radial glia-like morphology in the SGZ of the DG was considered as the total pool of NSCs of the DG, quiescent and recruited. NeuN+\BrdU+ cells in the GL of the DG were considered as mature neurons. For each animal from 5 to 20 sections of the left and right hemispheres were analyzed depending on the total cell count and anatomical size.

### 4.5. Statistical Analysis

Statistical analysis was performed using the Statistica 10.0 software (StatSoft Inc., Tulsa, OK, USA). Group differences in neurological scores were assessed using a non-parametric Mann-Whitney U test. Mean values and standard errors of mean (SEM) for each type of labeled cells were calculated in all hippocampal regions. For NeuN and Iba1 labeling, the density of NeuN+ cells per 100 × 100 µm^2^ was calculated. For other types of labeling, mean values and SE were calculated per section. Cell counts for each type of labeling were compared between the sham-operated group, positive controls, and fluoxetine-treated animals using a two-way factorial ANOVA (two factors: “group” with three levels, “time point” with two levels) followed by post-hoc analysis with Bonferroni’s correction.

The statistical significance for all the analyses was defined as a *p* value less than 0.05.

## 5. Conclusions

In summary, our data demonstrate that fluoxetine treatment within 10 days after GCI decreased early and long-term neuronal loss and inflammation, improved survival and functional recovery of animals, enhanced neurogenesis and prevented an early pathological increase in neural stem cell recruitment in the SGZ without ablating mature neuron production in 30 days after GCI. Taken together with the literature evidence of the efficacy of fluoxetine as a neuroprotective, anti-inflammatory and neurorestorative agent, this study suggests that fluoxetine may provide a promising therapy in cerebral ischemia.

## Figures and Tables

**Table 1:** Animal survival and neurological scores after surgery ^1^.

Group	Total Number	Survival (%)		Neurological Scores	
		10 Days	30 Days	10 Days	30 Days
Sham-operated	10	100	100	0 ^##^	0 ^##^
Ischemia	27	40.7	37.0	5.0 (2–9) **	4 (1–5) **
Ischemia + Fluoxetine	18	61.1	61.1	3 (1–8) *	1 (3–0) ^#^

^1^ Significant differences compared with sham-operated animals: ** *p* < 0.01, * *p* < 0.05. Significant differences compared with positive controls (group “Ischemia”): ^##^ *p* < 0.01, ^#^ *p* < 0.05. Neurological scores are presented as median (range).

## Supplementary Materials

Supplementary materials can be found at www.mdpi.com/1422-0067/19/1/162/s1.

## Abbreviations

ANOVA	Analysis of variance
BrdU	5-Bromo-2′-deoxyuridine
CA1	Cornu Ammonis, area 1
CA2	Cornu Ammonis, area 2
CA3	Cornu Ammonis, area 3
CNS	Central nervous system
DAPI	4′,6-Diamidino-2-phenylindole
DCX	Doublecortin
DG	Dentate gyrus of the hippocampus
DNA	Desoxynucleic acid
GCI	Global cerebral ischemia
GFAP	Glial fibrillary acidic protein
GL	Granular layer dentate gyrus
MCAO	Middle cerebral artery occlusion
*n*	Number of variables
NeuN	Neuronal nuclei protein
NSC	Neural stem cells
*p*	Significance level
PBS	Phosphate buffer
r	Pearson’s correlation coefficient
ROI	Regions of interest
RSCF	Russian Science Foundation
SEM	Standard error of mean
SGZ	Subgranular zone of dentate gyrus
SVZ	Subventricular zone
