# Supplementary material for: Effects of Fluoxetine on Hippocampal Neurogenesis and Neuroprotection in the Model of Global Cerebral Ischemia in Rats
Source: Int J Mol Sci. 2018 Jan 5;19(1):162. doi: 10.3390/ijms19010162 (PMC5796111; doi:10.3390/ijms19010162)
Supplement: Supplementary file 1 [file ijms-19-00162-s001.pdf]

International Journal of *Molecular Sciences*

MDPI

Article

Effects of Fluoxetine on Hippocampal Neurogenesis and Neuroprotection in the Model of Global Cerebral Ischemia in Rats

Marina Khodanovich [1,*], Alena Kisel [1], Marina Kudabaeva [1], Galina Chernysheva [2], Vera Smolyakova [2], Elena Krutenkova [1], Irina Wasserlauf [1], Mark Plotnikov [2] and Vasily Yarnykh [1,3]

Supplementary Table S1. Comparison of immunohistochemical parameters in the experimental groups.

Labeling		Region	Group, F (*p*)	Post-hoc test, 10 days			Post-hoc test, 30 days		
				Sham-operated	Ischemia	Ischemia+ Fluoxetine	Sham-operated	Ischemia	Ischemia+ Fluoxetine
NeuN, BrdU	NeuN+ cells, per 100 × 100 μm	hilus	**6.46 (*p* = 0.005)**	9.66 ± 0.72	8.11 ± 0.81	10.58 ± 0.58	9.09 ± 0.54	7.11 ± 0.77	8.99 ± 0.29
		DG	0.44 (*p* = 0.64)	63.8 ± 2.36	74.6 ± 1.66	70.0 ± 2.44	66.8 ± 4.91	61.0 ± 4.49	60.1 ± 2.44
		CA1	**43.48 (*p* < 0.001)**	28.22 ± 0.61	**11.64 ± 3.44 (*p* < 0.001, S)**	**26.62 ± 2.09 (*p* < 0.001, I)**	30.07 ± 2.30	**5.85 ± 1.38 (*p* < 0.001, S)**	**16.27 ± 2.13 (*p* = 0.003, S; *p* = 0.04, I)**
		CA2	**26.96 (*p* < 0.001)**	26.58 ± 2.38	**12.52 ± 2.76 (*p* < 0.001, S)**	19.64 ± 0.77	21.04 ± 0.69	**9.44 ± 1.75 (*p* = 0.001, S)**	15.90 ± 1.17
		CA3	**15.99 (*p* < 0.001)**	15.3 ± 1.43	12.1 ± 1.29	16.0 ± 0.58	16.9 ± 0.72	**8.5 ± 1.40 (*p* < 0.001, S)**	13.4 ± 0.66
	BrdU+ cells, per section	SGZ	**19.11 (*p* < 0.001)**	11.87 ± 2.12	**48.70 ± 4.69 (*p* < 0.001, S)**	**12.89 ± 5.10 (*p* < 0.001, I)**	5.33 ± 2.19	4.15 ± 0.86	3.82 ± 0.57
		GL	**19.62 (*p* < 0.001)**	4.53 ± 1.97	**31.00 ± 4.16 (*p* < 0.001, S)**	**9.08 ± 3.03 (*p* < 0.001, I)**	8.43 ± 1.99	15.90 ± 3.75	7.76 ± 1.58

International Journal of *Molecular Sciences*

MDPI

		hilus	**13.35<br>($p < 0.001$)**	2.87 ± 1.24	**24.00 ± 5.88<br>($p < 0.001$, S)**	**2.17 ± 0.73<br>($p < 0.001$, I)**	3.10 ± 0.62	3.67 ± 0.49	3.15 ± 0.97
		CA1	**77.20<br>($p < 0.001$)**	2.93 ± 0.93	**91.90 ± 8.36<br>($p < 0.001$, S)**	**10.39 ± 2.97<br>($p < 0.001$, I)**	5.87 ± 0.95	**46.16 ± 10.93<br>($p < 0.001$, S)**	**8.70 ± 2.63<br>($p = 0.002$, I)**
		CA2	**17.68<br>($p < 0.001$)**	0.63 ± 0.37	**14.00 ± 4.16<br>($p < 0.001$, S)**	**0.89 ± 0.54<br>($p < 0.001$, I)**	1.10 ± 0.56	6.87 ± 1.96	0.60 ± 0.37
	NeuN+/BrdU+ cells, per section	SGZ	1.31<br>($p = 0.3$)	0.17 ± 0.17	0.04 ± 0.04	0.47 ± 0.33	0.80 ± 0.37	2.80 ± 1.36	0.67 ± 0.44
		GL	0.56<br>($p = 0.6$)	0.00 ± 0.00	0.28 ± 0.20	1.05 ± 0.61	10.0 ± 1.58	7.40 ± 2.16	8.93 ± 1.18
		hilus	2.59<br>($p = 0.1$)	0.00 ± 0.00	0.00 ± 0.00	0.00 ± 0.00	0.00 ± 0.00	0.80 ± 0.37	0.25 ± 0.19
		CA1	0.73<br>($p = 0.1$)	0.00 ± 0.00	0.20 ± 0.20	0.00 ± 0.00	1.00 ± 0.43	0.60 ± 0.24	0.23 ± 0.12
		CA2	2.18 ($p = 0.1$)	0.00 ± 0.00	0.13 ± 0.13	0.00 ± 0.00	0.08 ± 0.08	0.40 ± 0.24	0.05 ± 0.05
Iba1, BrdU	Iba1+ cells, per 100 × 100 μm	CA1	**45.49<br>($p < 0.001$)**	1.43 ± 0.24	**12.98 ± 2.06<br>($p < 0.001$, S)**	**2.38 ± 0.36<br>($p < 0.001$, I)**	1.50 ± 0.19	**8.30 ± 1.59<br>($p = 0.003$, S)**	**1.53 ± 0.11<br>($p = 0.003$, I)**
	Iba1+/BrdU+ cells, per section	CA1	**26.80<br>($p < 0.001$)**	3.25 ± 2.36	**100.0 ± 26.52<br>($p < 0.001$, S)**	**5.00 ± 3.06<br>($p < 0.001$, I)**	3.00 ± 2.39	**58.74 ± 11.11<br>($p = 0.045$, S)**	**3.75 ± 2.50<br>($p = 0.049$, I)**
	BrdU+ cells, per section	CA1	**23.59<br>($p < 0.001$)**	6.75 ± 2.55	**107.5 ± 26.98<br>($p < 0.001$, S)**	**22.50 ± 4.68<br>($p = 0.002$, I)**	11.25 ± 4.15	**73.44 ± 15.20<br>($p = 0.03$, S)**	**12.50 ± 3.95<br>($p = 0.04$, I)**
GFAP, BrdU	GFAP+ cells, per section	SGZ	**18.89<br>($p < 0.001$)**	183.5 ± 9.3	**131.3 ± 7.4<br>($p = 0.02$, S)**	**179.0 ± 11.8<br>($p = 0.04$, I)**	164.7 ± 9.4	**104.7 ± 7.0<br>($p = 0.005$, S)**	**159.0 ± 13.9<br>($p = 0.01$, I)**

International Journal of
Molecular Sciences

MDPI

	GFAP+/BrdU+ cells, per section	SGZ	**5.41 (p = 0.01)**	5.67 ± 0.78	**16.50 ± 3.67 (p = 0.03, S)**	**5.20 ± 2.46 (p = 0.01, I)**	2.45 ± 0.78	2.95 ± 1.07	2.11 ± 0.28
		GL	**4.31 (p = 0.03)**	2.92 ± 1.08	4.70 ± 1.70	1.40 ± 0.29	1.61 ± 0.20	6.15 ± 1.86	3.04 ± 0.78
DCX	DCX+ cells, per section	DG	3.19 (p = 0.06)	63.27 ± 1.72	**101.38 ± 9.92 (p = 0.04, S)**	80.16 ± 10.05	61.63 ± 1.89	39.67 ± 3.52	**84.35 ± 11.76 (p = 0.009, I)**

Data are results of a two-way factorial ANOVA: (1) F-criteria with *p* values in parentheses for the main effect of the group factor, (2) mean ± SEM values for the experimental groups with *p* values in parentheses for Bonferroni's correction of pairwise comparison of the groups at time points 10 days and 30 days after surgery, S – comparison with the sham-operated group, I – comparison with positive controls. Significant tests are highlighted in bold.
